# Dual-time-point FDG-PET/CT Imaging of Temporal Bone Chondroblastoma: A Report of Two Cases

**Published:** 2015

**Authors:** Akira Toriihara, Atsunobu Tsunoda, Akira Takemoto, Kazunori Kubota, Youichi Machida, Ukihide Tateishi

**Affiliations:** 1 Department of Diagnostic Radiology and Nuclear Medicine, Tokyo Medical and Dental University, Yushima, Bunkyo-ku, Tokyo, Japan; 2 Department of Otolaryngology, Tokyo Medical and Dental University, Yushima, Bunkyo-ku, Tokyo, Japan; 3 Department of Human Pathology, Tokyo Medical and Dental University, Yushima, Bunkyo-ku, Tokyo, Japan

**Keywords:** Dual-time-point FDG-PET/CT, MRI, Temporal bone chondroblastoma

## Abstract

Temporal bone chondroblastoma is an extremely rare benign bone tumor. We encountered two cases showing similar imaging findings on computed tomography (CT), magnetic resonance imaging (MRI), and dual-time-point ^18^F-fluorodeoxyglucose (^18^F-FDG) positron emission tomography (PET)/CT. In both cases, CT images revealed temporal bone defects and sclerotic changes around the tumor. Most parts of the tumor showed low signal intensity on T2-weighted MRI images and non-uniform enhancement on gadolinium contrast-enhanced T1-weighted images. No increase in signal intensity was noted in diffusion-weighted images. Dual-time-point PET/CT showed markedly elevated ^18^F-FDG uptake, which increased from the early to delayed phase. Nevertheless, immunohistochemical analysis of the resected tumor tissue revealed weak expression of glucose transporter-1 and hexokinase II in both tumors. Temporal bone tumors, showing markedly elevated ^18^F-FDG uptake, which increases from the early to delayed phase on PET/CT images, may be diagnosed as malignant bone tumors. Therefore, the differential diagnosis should include chondroblastoma in combination with its characteristic findings on CT and MRI.

## Introduction

Chondroblastoma is a rare benign bone tumor, accounting for approximately 1% of all primary bone tumors (1). Temporal bone chondroblastoma is extremely rare (2), although some cases have been reported, using images of plain radiography, computed tomography (CT), magnetic resonance imaging (MRI), bone scintigraphy, and single-time point positron emission tomography (PET) using 2-deoxy-2-[^18^F]fluoro-D-glucose (^18^F-FDG) (2-4).

However, to the best of our knowledge, there have been no reports on the features of this tumor, based on dual-time-point ^18^F-FDG PET/CT images. Herein, we present two cases of temporal bone chondroblastoma, showing markedly elevated ^18^F-FDG uptake which increased from the early to delayed phase on PET/CT images, suggesting the need for caution against the misdiagnosis of these tumors as malignant tumors.

## Case report

### 

#### Case 1

A 32-year-old male patient presented with hearing loss in the left ear. MRI was performed at a hospital to explore the cause of patient’s symptoms. As the images indicated, a left temporal bone tumor was suspected, and the patient was referred to our hospital for further evaluation and treatment.

**Figure 1:**
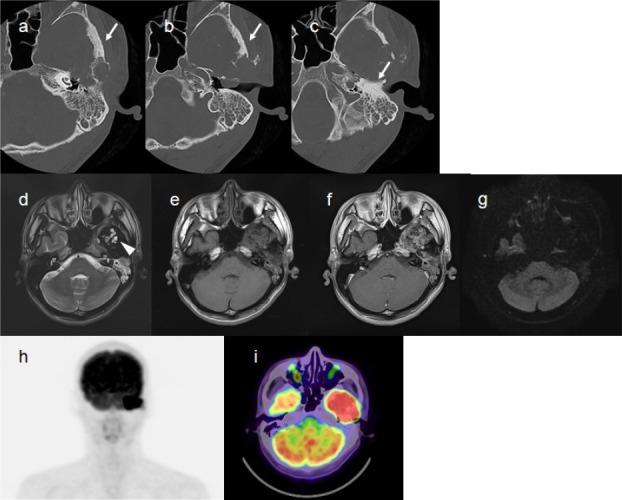
Imaging findings of case number 1. Bone-window CT images (a-c) show temporal bone defects and sclerotic changes (white arrows) around the tumor. T2-weighted MRI image (d) shows low signal intensity in most parts, with some areas of high signal intensity (white arrowhead). Plain T1-weighted image (e) and gadolinium contrast-enhanced T1-weighted image (f) show non-uniform enhancement. No elevated signal intensity is noted on the diffusion-weighted image (g). ^18^F-FDG PET/CT (h, maximum-intensity-projection image; i, fused PET/CT image at the delayed phase) shows markedly elevated uptake in the tumor. SUV_max_ values in the early phase (1h) and the delayed phase (2h) were 17.1 and 20.2, respectively; the RI was 18.1%

The patient underwent CT, MRI, and dual-time-point ^18^F-FDG PET/CT imaging at our hospital (Figure 1). The CT scan revealed temporal bone defects and secondary otitis media, caused by the tumor; sclerotic changes were observed in the surrounding bones (Figure 1 a-c). On T2-weighted MRI images, most of the tumor showed low signal intensity, while some components showed high signal intensity (Figure 1d).

Gadolinium contrast-enhanced T1-weighted images revealed non-uniform enhancement (Figure 1f). No increase in signal intensity was noted on diffusion-weighted images (Figure 1g). The tumor showed markedly elevated FDG uptake on ^18^F-FDG PET/CT images (Figures 1h and 1i). The maximum standardized uptake values (SUV_max_) in the early (1h) and delayed phases (2h) were 17.1 and 20.2, respectively.

The retention index (RI) was estimated at 18.1%, using the following formula:

RI=(delayed SUV_max_-early SUV_max_)/early SUV_max_×100 (%)

**Figure 2:**
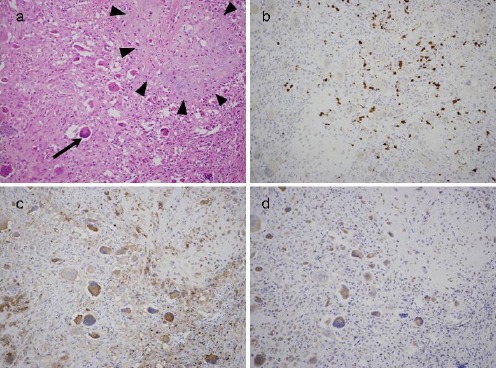
Histopathologic findings of case number 1. Examination of hematoxylin and eosin-stained sections (a) reveals that the tumor contains osteoclast-like, multinucleated giant cells (black arrow) and some chondroid matrices (surrounded by black arrowheads). In addition, hemosiderin deposition was observed broadly in the tumor. Immunohistochemical staining shows S-100 positive cells in the chondroid matrix (b). On the other hand, the expressions of Glut-1 (c) and HK-II (d) are weak

The microscopic examination of biopsy specimens revealed that the tumor was mainly composed of uniform, round to polygonal cells and numerous randomly-distributed osteoclast-type giant cells. Considering the tumor site, the preoperative diagnosis was giant cell reparative granuloma, and complete tumor resection was performed. Histopathologic examination of the resected specimen revealed that the tumor contained a chondroid matrix and hemosiderin deposition, which suggested tumor hemorrhage (Figure 2a).

Few inflammatory cells, including lymphocytes, were infiltrated in the tumor tissue. Immunohistochemical analysis revealed S-100 positive cells (Figure 2b). Considering the obtained results, the tumor was diagnosed as a temporal bone chondroblastoma. Although these evaluations are not routinely applied, further analyses revealed weak expressions of both glucose transporter-1 (Glut-1) and hexokinase II (HK-II) (Figures 2c and 2d).

#### Case 2

**Figure 3:**
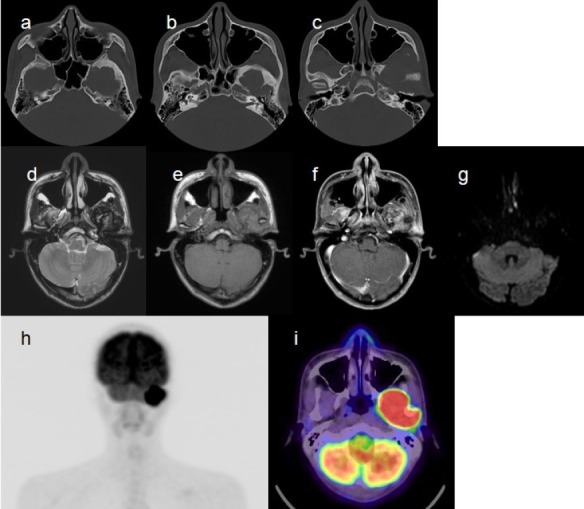
Imaging findings of case number 2. Bone-window CT images (a-c), MRI (d, T2-weighted image; e, T1-weighted image; f, gadolinium contrast-enhanced T1 weighted image; g, diffusion-weighted image), and ^18^F-FDG PET/CT (h, maximum-intensity-projection image; i, fused PET/CT image at the delayed phase) are presented. The findings are similar to those of case 1. SUV_max_ values in the early phase (1h) and the delayed phase (2h) were 17.3 and 19.4, respectively; the RI was 12.1%

A 22-year-old male patient had undergone myringotomy several times within the previous year for recurrent otitis media with effusion in the left ear. MRI was performed at another hospital to explore the cause of recurrent episodes, and left temporal bone tumor was discovered. The patient underwent CT, MRI, and dual-time-point ^18^F-FDG PET/CT at our hospital (Figure 3).

The findings of these imaging modalities were quite similar to those reported in case 1. However, in this case, signal intensity within the tumor in T2-weighted images was slightly lower than that reported in case 1. The SUV_max_ values in the early and delayed phases on ^18^F-FDG PET/CT images were 17.3 and 19.4, respectively, and the RI was 12.1%. Tumor biopsy was performed. The preoperative diagnosis was a giant cell tumor, based on which the tumor was completely removed. The histopathological findings were similar to those in case 1 and the final diagnosis was temporal bone chondroblastoma.

## Discussion

Chondroblastoma is a rare, benign, cartilage-producing neoplasm, which is typically composed of round or polygonal chondroblasts, osteoclast-type giant cells, and a bluish to eosinophilic chondroid intracellular matrix with focal calcification (7). This tumor predominantly arises from the epiphyses of long bones. Only around 80 cases of temporal bone chondroblastoma have been reported (2).

It is known that chondroblastoma often shows increased ^18^F-FDG uptake in spite of its benign pathological features (6). Hamada et al. reported the case of an ischial chondroblastoma with increased ^18^F-FDG uptake (7). Interestingly, in the mentioned cases and our two patients, immunohistochemical analysis demonstrated only weak expression of Glut-1 and HK-II. Red blood cells were properly marked, though other cell types were not stained.

Few lymphocytes were scattered; thus, inflammation is unlikely to be the cause of FDG accumulation. The precise mechanism of increased ^18^F-FDG uptake in chondroblastoma is still unknown. However, considering the low signal intensity in T2-weighted MRI images and hemosiderin deposition in the resected specimen, tumor hemorrhage may contribute to some ^18^F-FDG pooling.

On the other hand, there have been no reports evaluating the changes in ^18^F-FDG uptake from the early to the delayed phase in chondroblastoma. However, Tian et al. indicated that dual-time-point ^18^F-FDG PET may be helpful for differentiating malignant from benign bone tumors (8); it should be mentioned that patients with chondroblastoma were not included in their study. They showed that the sensitivity, specificity, and accuracy of ^18^F-FDG PET for such discrimination were 90.6%, 76.0%, and 83.7%, respectively, when 10% RI was considered as the cut-off point.

Our two presented cases would be labeled false-positive if the mentioned criteria were to be adopted. We should bear in mind that the ^18^F-FDG uptake, which increases from the early to the delayed phase in chondroblastoma, can result in misdiagnosis of this tumor as a malignancy.

Temporal bone chondroblastoma appears as a well-defined osteolytic mass with mild enhancement on CT images. Calcification within the lesion has been documented in 20-50% of cases (3). On MRI, chondroblastoma is generally visualized as low signal intensity on T1-weighted images and as low-to-high signal intensity on T2-weighted images. Gadolinium-enhanced MRI reveals heterogeneous enhancement of the solid parts of the tumor. Some tumors also contain cystic components (4).

To the best of our knowledge, the features of these tumors on diffusion-weighted images have not been reported. Based on the findings in our case report, chondroblastoma is not likely to show elevated signal intensity on diffusion-weighted images.

Our two cases were preoperatively diagnosed as giant cell reparative granuloma and giant cell tumor, respectively. Osteoclast-like, multinucleated giant cells are histological common findings in chondroblastoma, giant cell reparative granuloma, and giant-cell tumors (5, 9, 10). Since the biopsy specimens in our cases did not reveal the presence of a chondroid matrix, accurate diagnosis was difficult at that time.

In the surgical specimen, in addition to the detection of a chondroid matrix, immunohistochemical analysis of S-100 expression assisted us in making the final diagnosis of chondroblastoma. Demonstration of S-100 positivity in chondroblasts is helpful in differentiating chondroblastoma from giant cell reparative granuloma and giant cell tumors (5, 10).

To the best of our knowledge, there have been no reports indicating ^18^F-FDG PET/CT findings of giant cell reparative granuloma. On the other hand, giant cell tumors are known to show markedly elevated ^18^F-FDG uptake (6, 8). In fact, it is difficult to discriminate between chondroblastoma and giant cell tumors, based on the level of ^18^F-FDG uptake.

In conclusion, we reported two cases of temporal bone chondroblastoma. Based on the findings, when temporal bone tumors with markedly elevated ^18^F-FDG uptake, which increases from the early to the delayed phase on PET/CT images, are encountered, chondroblastoma with its specific CT and MRI features should be considered in the differential diagnosis.

## Conflicts of interest

No potential conflicts of interest were disclosed.
